# Retraction Note: *P16*^*INK4a*^ deletion ameliorated renal tubulointerstitial injury in a stress-induced premature senescence model of *Bmi*-1 deficiency

**DOI:** 10.1038/s41598-025-85803-8

**Published:** 2025-01-14

**Authors:** Jianliang Jin, Jianguo Tao, Xin Gu, Zhenzhen Yu, Rong Wang, Guoping Zuo, Qing Li, Xianhui Lv, Dengshun Miao

**Affiliations:** 1 The State Key Laboratory of Reproductive Medicine; Key Laboratory for Aging & Disease, Research Centre for Bone and Stem Cells, Department of Human Anatomy, Nanjing Medical University, Nanjing, Jiangsu 211166 China; 2 Laboratory Centre for Basic Medical Sciences, Nanjing Medical University, Nanjing, Jiangsu 211166 China; 3 Department of Science and Technology, Jiangsu Jiankang Vocational College, Nanjing, Jiangsu 210029 China

Retraction of: *Scientific Reports* 10.1038/s41598-017-06868-8, published online 08 August 2017

The Editors have retracted this Article.

Concerns were raised regarding similar images between Figure 4 of this article, and Figure 2 in^1^ both representing WT medulla Bmi-1, and in Figure 3 (WT IL-1ß cortex), Figure 3 in^1^ (WT TNFα cortex). Within the Article, additional issues were found between Figures 2d WT 8-OHdG cortex, and 3c WT TNFα cortex, between Figures 2d cortex WT 8-OHdG and 3d cortex WT NF-$\upkappa$B-p65, between Figures 2d medulla WT 8-OHdG and 3d medulla WT NF-$\upkappa$B-p65 and between Figures 2e medulla WT CD3 and 2f cortex WT F4/80. There also appears to be similarity between the ß-actin control bands in Figures 3 and 4. The Authors provided original images and repeats, but the data provided did not address the concerns. The Editors no longer have confidence in the data underlying this Article.

Authors Dengshun Miao has informed the publisher that author Xianhui Lv is deceased. Authors Dengshun Miao, Rong Wang, Zhenzhen Yu, Jianliang Jin, Xin Gu and Jianguo Tao disagree with the retraction. Authors Guoping Zuo and Qing Li did not respond to correspondence regarding this retraction.
